# Ovarian Rev-erbα: a central regulator of fertility following chronodisruption

**DOI:** 10.3389/fendo.2026.1742988

**Published:** 2026-02-18

**Authors:** Shalini Gupta, Saumyata Kumawat, Vipashu Kaushal, Rashmi Arora, Sumit Kumar, Rahul Sharma, Anshu Bhardwaj, Neeraj Khatri, Pawan Gupta

**Affiliations:** 1 Department of Molecular Biology, Council of Scientific and Industrial Research, Institute of Microbial Technology, Chandigarh, India; 2 Academy of Scientific and Innovative Research (AcSIR), Ghaziabad, Uttar Pradesh, India

**Keywords:** clock genes, folliculogenesis, REV-ERBα, superovulation, therapeutic

## Abstract

**Introduction:**

Circadian rhythm disruption caused by shift work, nutritional imbalance, and the stresses of modern life can alter hormone secretion, lead to menstrual irregularities, impair follicle development, and contribute to ovarian hypoplasia. The mechanistic role of circadian rhythm disruption in reproductive disorders has been thoroughly investigated. Nuclear receptors are known to play an important role in female reproduction and in maintaining pregnancy. Rev-erbα, a circadian nuclear receptor, is a key component of the circadian clock and helps sustain circadian rhythm.

**Materials and Methods:**

We evaluated the potential prophylactic and therapeutic functions of Rev-erbα in supporting female fertility by orchestrating a series of events that culminate in successful pregnancy. We generated a circadian rhythm-disrupted female mouse model to study fertility.

**Results:**

The Rev-erbα ligand SR9011 improved the fertility index in these circadian rhythm-disrupted female mice. Moreover, SR9011 treatment restored impaired ovarian follicular cell proliferation and division, regulated steroidogenesis and steroid hormone production, enhanced progesterone and melatonin secretion, and mitigated the adverse effects of circadian disruption on folliculogenesis. SR9011 also reduced follicular atresia and promoted follicle development in CR-disrupted mice. Rev-erbα is a key regulator influencing oocyte retrieval, as demonstrated in mice treated with the Rev-erbα antagonist SR8278.

**Discussion/Conclusion:**

These findings suggest that targeting Rev-erbα signaling and elucidating its mechanistic role in female reproduction could inform the development of more effective strategies for treating female infertility. As a sought-after druggable target, Rev-erbα has a broad range of potential therapeutic applications and has recently attracted considerable attention in the context of female infertility treatment.

## Graphical Abstract

**Figure:**
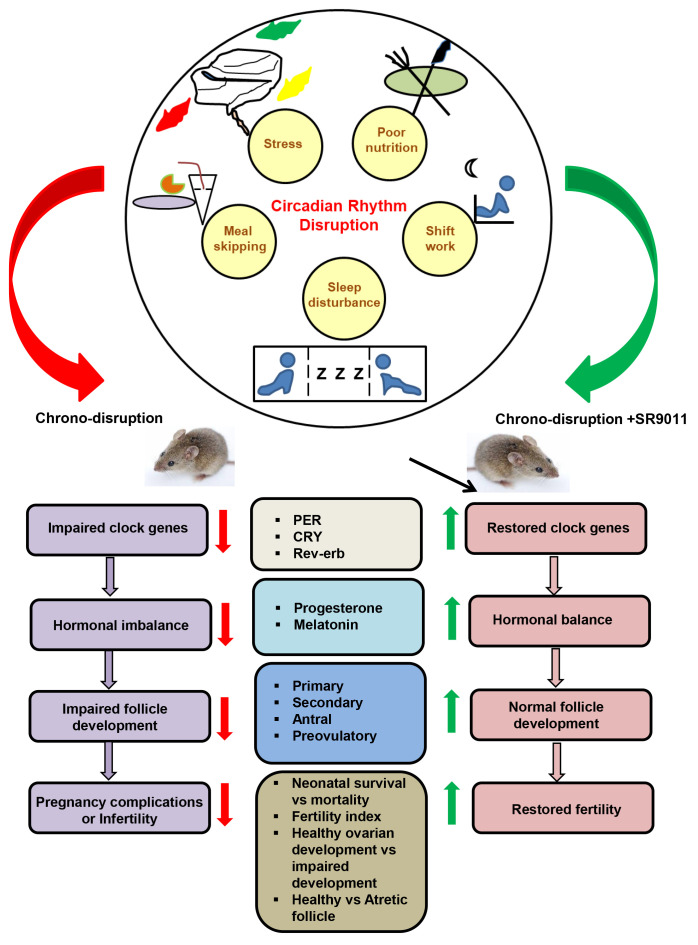

## Introduction

Infertility is a global health concern that arises from compromised endometrial regeneration and poor uterine receptivity. It affects millions of women of reproductive age and poses a significant threat to the human species reproduction (1). Other common causes include ovulatory dysfunction, polycystic ovarian syndrome, blockage of the fallopian tubes, endometriosis, infections, and endocrine disorders that disrupt hormone balance. Moreover, circadian rhythm (CR) disruptions, such as those caused by shift work and nutritional imbalances, have been associated with reduced fertility (2). Hormone secretion is also affected by stress related to modern lifestyles, further contributing to infertility (2).

Menstrual and pregnancy abnormalities in female shift workers highlight the importance of CR in women’s reproductive health (3). Sleep disruption may alter the timing of reproductive hormone secretion. The interaction between disturbed sleep and psychological distress in female reproduction has not been adequately addressed and may represent an imperative factor to consider during the investigation and treatment of female infertility. These physiological processes are controlled by core clock genes present in every cell of the human body and regulated by the “master clock” in the suprachiasmatic nucleus (SCN). Clock genes in the SCN synchronize peripheral clocks, although peripheral clocks can be influenced by factors other than the central clock. For instance, sleep disturbance, an unhealthy diet, or abnormal feeding patterns can cause the peripheral clock (i.e., in the liver and intestine) to become misaligned with the central clock (4). Many reproductive processes are strongly circadian, and intrinsic rhythmicity within reproductive tissues has been demonstrated. The SCN regulates peripheral cell rhythms through the autonomic nervous system and communicates with the pineal gland to control the cyclic melatonin production. In addition to maintaining circadian rhythm, melatonin also synchronizes the ovary’s internal circadian clock and modulates key reproductive processes, including ovulation, regulation of the menstrual cycle, and fertility. Evidence suggests its involvement in the pathophysiology of reproductive conditions such as polycystic ovary syndrome and endometriosis (PCOS), where its antioxidant and anti-inflammatory actions may improve ovarian function and fertility (5). It regulates clock gene expression, enhances mitochondrial protein levels, and modulates pyroptosis in PCOS ovaries (6). Moreover, it promotes follicular development by increasing VEGF expression in secondary follicles, thereby stimulating follicular angiogenesis, a critical process for follicular growth and maturation (7). Its levels must be tightly regulated to restore fertility outcomes compromised by circadian rhythm disruption.

The increased prevalence of disease associated with CR disruption underscores the need to better understand how CR disruption can contribute to pregnancy complications.

Nuclear receptors (NRs) play diverse roles in female reproduction and in maintaining successful pregnancies. Implantation of the blastocyst in the uterus is regulated by the ovarian hormones progesterone and estrogen. These pregnancy-related hormones act via their NRs to regulate the transcription of the genes involved in endometrial function (8). The NR vitamin D receptor has also been recognized as essential for granulosa cell differentiation, while liver receptor homolog 1 has been reported to play a key role in mouse fertility (9), ovulation, and ovarian steroidogenesis (8, 10). Retinoic acid receptor is involved in early embryonic development (11). Chicken ovalbumin upstream promoter transcription factor II is required for placental development and angiogenesis (12, 13). Steroidogenic factor 1 plays a critical role in folliculogenesis and ovulation, and its absence in granulosa cells leads to impaired ovulation (14, 15). Androgen receptor signaling is crucial for endometrial function, whereas its disruption results in compromised reproductive outcomes (16). Liver X receptor regulates ovarian exocrine and endocrine function, as well as uterine contractility (17). Vitamin D receptor expression increases during pregnancy and supports reproductive function (18). Vitamin D is involved in folliculogenesis, cell differentiation, luteinization, and the regulation of steroidogenesis (19). Collectively, NRs are essential for female fertility, and their dysregulation can lead to pregnancy complications.

NRs are therapeutic targets for numerous pathological conditions associated with CR disruptions (20). Rev-erbα stabilizes the molecular clock and directly regulates clock genes and has been effectively targeted with small-molecule ligands. Rev-erbα KO mice exhibit altered sleep homeostasis (21), and a synthetic Rev-erb agonist alters sleep architecture (22). Moreover, Rev-erbα has been reported to regulate circadian drug metabolism, with important implications for chronopharmacology (23).

Dysregulation of Rev-erbα has a significant impact on circadian rhythm; however, its effect on female fertility has not been fully addressed. Here, we demonstrate for the first time the function of Rev-erbα in folliculogenesis, steroidogenesis, luteinization, and gonadotropin-induced oocyte retrieval. Rev-erbα promotes follicle development by facilitating oocyte maturation and release and by regulating hormone secretion, ultimately contributing to fertility restoration. Rev-erbα ligand SR9011 prevents primary ovarian insufficiency and supports ovarian cell development. In a superovulation experiment, we observed that Rev-erbα is a key factor driving oocyte release during circadian rhythm disruption, and inhibition of Rev-erbα with SR8278 affects oocyte release. In conclusion, we provide insight into the significant effects of Rev-erbα ligand on female fertility, encompassing follicular development, oocyte release, and pregnancy.

## Materials and methods

### Ethics and animals

C57BL/6 male and female mice were obtained from Jackson Laboratories. Mice aged 4 to 6 weeks were housed in the mouse facility at the Institute of Microbial Technology (IMTECH). For CR disruption, female mice were initially placed under a 12-h light/dark (L/D) cycle. After 1 week of activity recording in the LD cycle, the mice were placed in constant darkness for 4–6 weeks to induce CR disruption. Experimental mice were 6–8 weeks old at the time of use. A total of 132 female mice were included in the study. Mice were euthanized by cervical dislocation without anesthesia. All experimental procedures were authorized by the Institutional Animal Ethics Committee and conducted in accordance with national regulatory guidelines (No. 55/1999/CPCSEA), Ministry of Environment and Forests, Government of India.

### Experimental design for SR9011 dosage

1. Prophylactic treatment: SR9011 (100 mg/kg) was administered once per week during the CR disruption period, and samples were processed 24 h after each treatment over a period of 4 weeks.
2. Therapeutic treatment: SR9011 (100 mg/kg) was administered for five consecutive days after 4 weeks of CR disruption, and samples were collected 1 week later.

### Fertility study and reproductive performance

For fertility evaluation, control female and CR-disrupted female mice, with or without SR9011 treatment, were paired with normal male mice (monogamous mating). CR disruption and SR9011 administration were applied both during and after CR disruption to evaluate their prophylactic and therapeutic effects on fertility. Mating behavior was observed under a normal 12-h L/D cycle. Breeding data were generated following the method described by Handelsman et al. (24), with modifications detailed in the Materials and methods section. Pup counts and body weight measurements were recorded. Breeding data were analyzed using group-specific endpoints reflecting the cessation of breeding activity for each group. This approach was chosen to account for natural differences in reproductive performance between groups under their respective conditions.

### Quantitative real-time PCR

Total RNA was extracted from the ovaries using the TRIzol method (Ambion, Invitrogen, Massachusetts, USA). Using 1 µg of RNA, complementary DNA (cDNA) was synthesized with the Verso cDNA Synthesis Kit (Thermo Fisher Scientific, Massachusetts, USA) following the manufacturer’s protocol. cDNA amplification was performed using the Dynamo ColorFlash SYBR Green Kit (Thermo Fisher Scientific). Relative fold change was calculated using the 2^−ΔΔCt^ method.

The primer sequences used for qRT-PCR are as follows:

*Per2* Forward: 5′-CAGGCTGAGTTCCCTAGTCG-3′, Reverse: 5′-TGTGCAGTCCAGACCAGAAG-3′; *Cry1* Forward: 5′-GTGGATCAGCTGGGAAGAAG-3′, Reverse: 5′-CACAGGGCAGTAGCAGTGAA-3′; *Fshr* Forward: 5′-TGATGTTTTCCAGGGAGCCT-3′, Reverse: 5′-CTGGCCTCAATGAGCATGAC-3′; *Star* Forward: 5′-TTGGGCATACTCAACAACCA-3′, Reverse: 5′-GAAACACCTTGCCCACATCT-3′; *Amh* Forward: 5′-GGGAGACTGGAGAACAGCAG-3′, Reverse 5′-GTCCACGGTTAGCACCAAAT-3′; *Cyp11a1* Forward: 5′-CACAGACGCATCAAGCAGCAAAA-3′, Reverse: 5′-GCATTGATGAACCGCTGGGC-3′; and *Actin* Forward: 5′-ATTTCTGAATGGCCCAGGTC-3′, Reverse: 5′-GTCTCAAGTCAGTGTACAGGC-3′.

### Western blotting

The ovary was homogenized, and cell lysates were prepared. Protein concentrations were determined using Bradford reagent (Sigma-Aldrich, Darmstadt, Germany). Proteins were separated by 10% sodium dodecyl sulfate–polyacrylamide gel electrophoresis and transferred onto a polyvinylidene difluoride membrane (Immobilon-P; Millipore, Darmstadt, Germany). Membranes were blocked in 1 × TBS containing 0.1% Tween 20 and 5% skim milk (Merck Millipore) prior to incubation with the following primary antibodies: rabbit anti-CYP11A1 (1:1,000, Affinity Biosciences company/manufacturer's location Cincinnati, USA), rabbit anti-STAR (1:1,000, Affinity Biosciences), rabbit anti-follicle-stimulating hormone receptor (FSHR; 1:1,000, Affinity Biosciences, Cincinnati, USA), rabbit anti-PER2 (1:1,000, Affinity Biosciences), rabbit anti-CRY1 (1:1,000, Affinity Biosciences), rabbit anti-p27 (1:1,000, Affinity Biosciences), and rabbit anti-CYCLIN D2 (1:1,000, Affinity biosciences). The PVDF membrane was then incubated with Horseradish Peroxidase (HRP)-conjugated secondary antibodies (1:2,000, Abcam, Cambridge, UK) for 1 h at room temperature (RT) and detected using chemiluminescent HRP substrate Luminata Forte (Millipore).

### Tissue preparation

Mice were killed, and ovaries were collected. For immunohistochemistry, ovaries were fixed in 10% formalin at RT. For RNA analysis, ovaries were stored in RNAlater for total RNA isolation. For protein analysis, ovaries were frozen in liquid nitrogen and stored at – 80°C until use for Western blotting.

### Immunohistochemistry

Ovarian sections were incubated overnight at 4 °C with rabbit anti-Ki67 and rabbit anti-p27 antibodies (1:100, Affinity Biosciences), followed by a 1 h incubation at RT with a biotinylated secondary antibody. After washing with PBS, sections were treated with diaminobenzidine (DAB) and H_2_O_2_ as a chromogen and then submerged in water to stop the reaction. Sections were counterstained with Cole’s hematoxylin for 1–2 min, air-dried, cleaned in xylene, and mounted with DPX. Images were captured using a light microscope.

### Histology analysis and follicle counting

Ovaries from the control and treated groups were extracted and fixed in 10% formalin for 24 h at RT. The fixed samples were then dehydrated, embedded in paraffin blocks, and sectioned using a rotary microtome. Ovary sections were cut at 6 μm thickness, stained with hematoxylin and eosin (H&E), and mounted on slides. For follicle counting, serial sections from each ovary were placed on glass slides in order and stained with H&E. The first section was selected randomly, and only follicles with clearly visible nuclei were scored. Follicles were counted in every fifth section throughout the ovary to avoid double-counting, as individual follicles span multiple consecutive sections. To estimate total follicle numbers, the counted follicles in each category (primordial, primary, secondary, antral, preovulatory, and atretic) were multiplied by the section interval 5 and section thickness (6 µm), following the approach described by Hirshfield et al. (25, 26). This systematic sampling strategy provides a representative estimate of total follicle numbers while accounting for tissue depth and section sampling. Stained sections were analyzed at different developmental stages using a Nikon ECLIPSE E 600 light microscope equipped with an E66 digital camera (Nikon, Tokyo, Japan).

### Morphological investigation of follicles in ovarian sections

Ovarian follicles were categorized into different stages based on their morphology: primordial, primary, secondary, preovulatory, ovulatory, and atretic follicles. A follicle containing an oocyte surrounded by a single layer of squamous granulosa cells was identified as a primordial follicle. Primary follicles consisted of oocytes enclosed by a single layer of cuboidal granulosa cells, whereas secondary follicles were surrounded by multiple layers of granulosa cells. Follicles with five or more granulosa cell layers were classified as antral follicles, which typically contained one or two small antral fluid spaces. Follicles exhibiting cells in the zona pellucida were categorized as atretic follicles.

### Superovulation

Mice aged 6–8 weeks were treated intraperitoneally (i.p.) with 5 IU of PMSG (Sigma-Aldrich) followed 48 h later by 5 IU of Human Chorionic Gonadotropin (hCG) (Sigma-Aldrich) via i.p. injection. Mice were euthanized 12–16 h after hCG administration, and oocytes were collected from the oviducts. Oocytes were counted manually under a stereomicroscope.

### Hormone assays

Mice were killed after 4–6 weeks, and blood was collected by cardiac puncture into microcentrifuge tubes. After centrifugation, serum was stored at – 80°C until analysis. Hormone assays for progesterone and melatonin (Elabscience, Houston, USA) were performed on the serum samples according to the manufacturer’s instructions.

### Statistical analysis

GraphPad Prism software was used for all statistical analyses. Data are presented as either the mean ± standard error of the mean (SEM) for measurements reflecting precision across images or replicates, or as the mean ± standard deviation (SD) for measurements reflecting biological variability across animals. All experiments were performed in triplicate. Graphs were prepared with GraphPad Prism. Statistical differences between groups were assessed using a two-tailed unpaired *t*-test with Welch’s correction. Differences were considered statistically significant at ^*^p < 0.05 or ^*^p = 0.05, ^**^p < 0.01, ^***^p < 0.001, or ^****^p  < 0.0001.

## Results

### Reproductive performance through breeding

**Figure 1:**
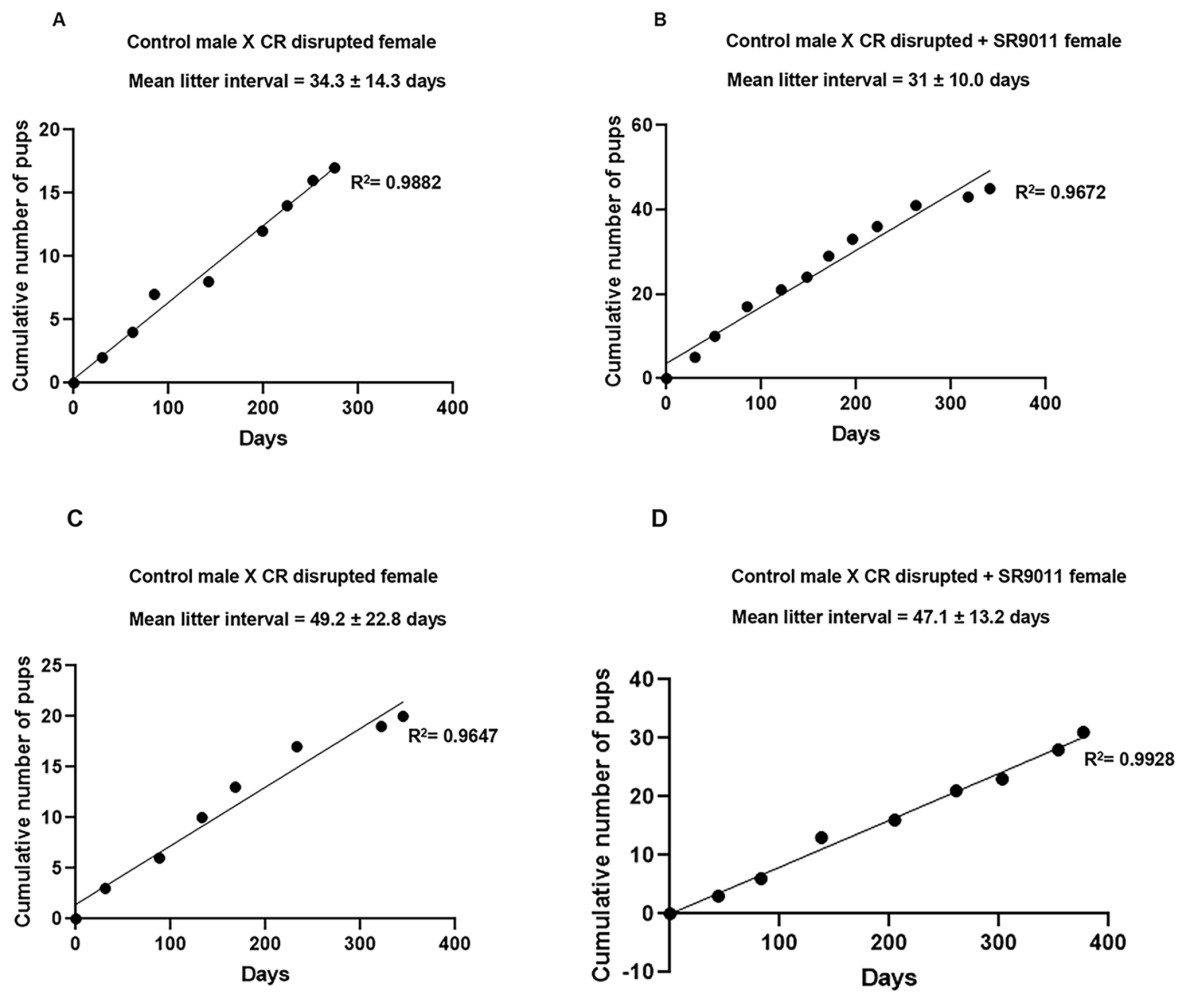
Effect of Rev-erbα on fertility. Breeding efficiency was assessed by examining the impact of circadian rhythm disruption on fertility outcomes, and the effectiveness of SR9011 on breeding was evaluated using both prophylactic **(A, B)** and therapeutic **(C, D)** approaches. Each litter is indicated on the *x*-axis according to the number of days elapsed since the mating trial commenced. At the occurrence of each litter, the plot exhibits a vertical shift corresponding to the cumulative number of offspring produced. The graph presents data from individual cages, illustrating the following outcomes: **(A)** CR-disrupted mouse pairs produced nine live litters over 275 days. The effectiveness of SR9011 on breeding was assessed, revealing that **(B)** CR-disrupted mice treated with SR9011 produced 12 live litters over 341 days, whereas **(C)** untreated CR-disrupted mouse pairs produced eight live litters over 345 days. **(D)** CR-disrupted pairs treated with SR9011 produced nine live litters over 377 days. The mean litter interval is presented as the mean time interval between litters ± SD.

The mouse is a valuable animal model for studying mammalian reproductive physiology due to its short reproductive cycle, high breeding efficiency, and small size with low maintenance costs. We generated a CR-disrupted female mouse model to study fertility. To evaluate reproductive performance, CR-disrupted female mice, treated with or without SR9011, were bred with normal male mice, while control female mice were bred with normal male mice (control pair). Breeding experiments were conducted to assess both the prophylactic and therapeutic efficacy of SR9011 in restoring fertility following CR disruption. For evaluation of the prophylactic effect, mice received SR9011 treatment during the period of CR disruption. We observed that the live litter size was larger in control pairs, with more frequent breeding cycles (**Supplementary Figure S1A**), compared with CR-disrupted mouse pairs (**Figure 1A**; **Supplementary Figure S1B**), indicating that CR disruption adversely affects reproductive performance. In SR9011-treated pairs, live litter size was higher, and breeding cycles were more consistent than in pairs subjected to CR disruption alone (**Figure 1B**). Similarly, when evaluating the therapeutic efficacy of SR9011, breeding efficiency was significantly higher in control pairs than in CR-disrupted pairs (**Supplementary Figures S1C, D**). The breeding efficiency of the CR-disrupted pairs treated with SR9011 showed a marked improvement compared with CR disruption alone (**Figures 1C, D**), suggesting that SR9011 restores fertility impaired by CR disruption (**Figure 1D**). Pup body weight was higher in control pairs than in CR-disrupted pairs; however, no significant difference in pup weight was observed between CR-disrupted pairs treated with SR9011 and untreated CR-disrupted pairs.

We also recorded additional reproductive metrics. Mating latency, defined as the time from pairing to first successful litter, was prolonged in CR-disrupted females; control pairs produced their first litter approximately 52 days after pairing, whereas CR-disrupted pairs required ~ 90 days. Gestational/interlitter intervals were also extended in CR-disrupted mice (mean: 51.8 days) compared with controls (32.9 days) (**Supplementary Figures S1A, B**), indicating delayed reproductive cycling under circadian disruption. While evaluating the prophylactic effect of SR9011, CR-disrupted females and CR + SR9011 groups were paired for mating simultaneously (**Figures 1A, B**). CR-disrupted females produced their first litter approximately 30 days after mating, with a mean interlitter (gestational) interval of 34.3 days. Notably, CR + SR9011-treated females also produced pups at ~ 30 days but exhibited a shorter mean interlitter interval of 31 days, indicating improved reproductive efficiency following Rev-erbα activation.

In the therapeutic setting, control females produced their first litter at approximately 44 days, with a mean interlitter interval of 43.1 days. CR-disrupted females produced their first pups at ~ 31 days but exhibited a prolonged mean interlitter interval of 49.2 days. In contrast, CR + SR9011-treated females produced pups at ~ 44 days, with a reduced mean interlitter interval of 47.1 days compared with CR-disrupted mice, suggesting partial restoration of reproductive timing.

Although the pregnancy rate per estrous cycle was not specifically recorded, the overall pregnancy rate was reduced in CR-disrupted mice. Neonatal mortality was recorded separately across five breeding cages per condition but was not included in the cumulative litter plots.

Under CR disruption, neonatal mortality was elevated. In the preventive cohort, CR-disrupted pairs exhibited 12 neonatal deaths, whereas CR-disrupted pairs treated with SR9011 showed eight neonatal deaths; no neonatal deaths were observed in control pairs. Similarly, in the therapeutic cohort, CR-disrupted mice showed nine neonatal deaths, while CR + SR9011-treated mice exhibited six neonatal deaths, with no mortality observed in controls. Mortality of the female breeding pairs was also observed, further indicating the physiological stress associated with circadian disruption.

### Impact of CR disruption on clock gene expression

Using quantitative real-time PCR (qRT-PCR), we assessed the relative messenger RNA (mRNA) levels of *Rev-erbα*, Period (*Per2*), and Cryptochrome (*Cry1*) in the ovaries of both control and CR-disrupted mice (**Supplementary Figure S2**). Compared with controls, the expression of *Rev-erbα*, *Per2*, and *Cry1* was decreased in the ovaries of CR-disrupted mice, indicating that circadian disruption adversely affects ovarian clock gene expression. β-Actin was used as an internal control for normalization.

### Effect of SR9011 on steroidogenesis, folliculogenesis, clock genes, and follicular cell division

**Figure 2:**
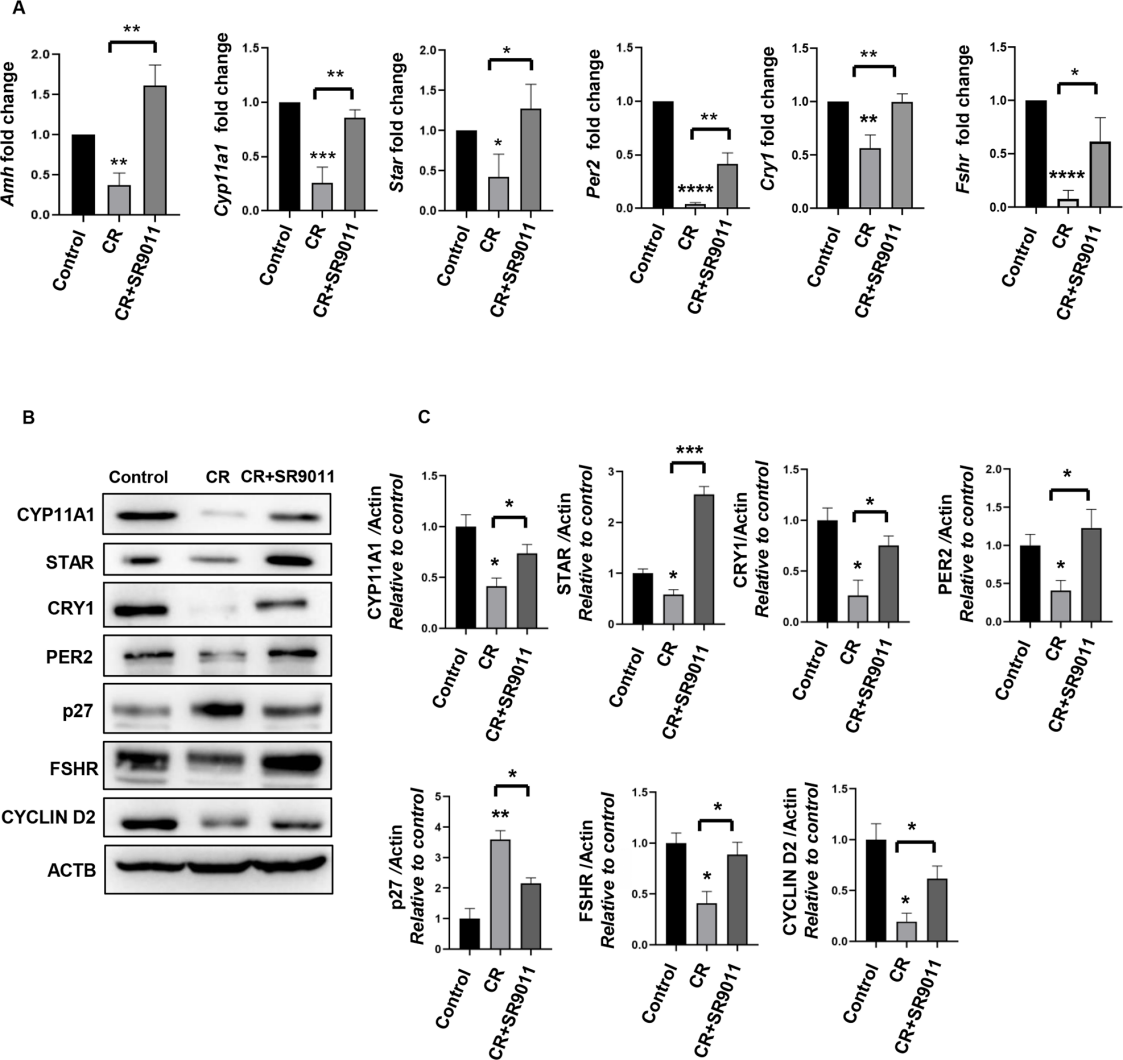
Prophylactic effect of Rev-erbα on clock genes, steroidogenesis, folliculogenesis, and cell division. The prophylactic effects of SR9011 **(A–C)** were evaluated. **(A)** The expression levels of *Amh*, *Star*, *Cyp11a1*, *Per2*, *Cry1*, and *FSHR* were analyzed in the ovaries of control mice, CR-disrupted mice, and CR-disrupted mice treated with SR9011 using qRT-PCR. **(B)** Western blotting was performed to examine the expression of CYP11A1, STAR, PER2, CRY1, FSHR, P27, and CYCLIN D2 in the ovaries of control mice, CR-disrupted mice, and CR-disrupted mice treated with SR9011. **(C)** Quantification of protein bands is shown. Asterisks represent significant differences compared with the control group or as indicated (^****^p < 0.0001, ^***^p < 0.001, ^**^p < 0.01, ^*^p < 0.05 or *p* = 0.05). Data are presented as the average **(A, C)** or as representative images **(B)** from three independent experiments. Results in **(A)** are expressed as mean ± SD, and results in **(C)** are expressed as mean ± SEM.

**Figure 3:**
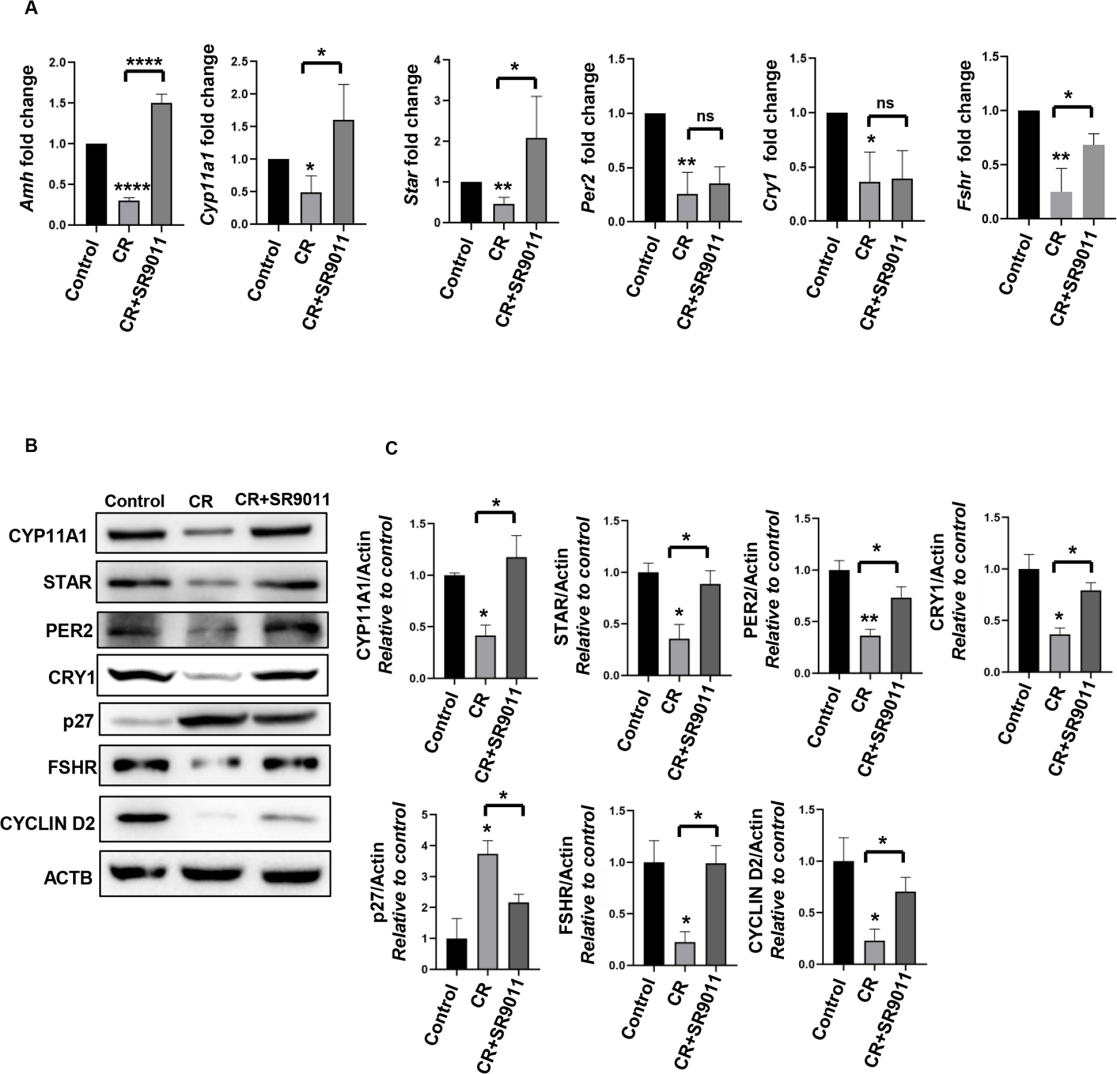
Therapeutic effect of Rev-erbα on clock genes, steroidogenesis, folliculogenesis, and cell division. The therapeutic effects of SR9011 **(A–C)** were evaluated. **(A)** The expression levels of *Amh*, *Star*, *Cyp11a1*, *Per2*, *Cry1*, and *FSHR* were analyzed in the ovaries of control mice, CR-disrupted mice, and CR-disrupted mice treated with SR9011 using qRT-PCR. **(B)** Western blotting was performed to examine the expression of CYP11A1, STAR, PER2, CRY1, FSHR, P27, and CYCLIN D2 in the ovaries of control mice, CR-disrupted mice, and CR-disrupted mice treated with SR9011. **(C)** Quantification of protein bands is shown. Asterisks represent significant differences compared with the control or as indicated (^****^p < 0.0001, ^**^p < 0.01, ^*^p < 0.05 or *p* = 0.05). Data are presented as the average **(A, C)** or as representative images **(B)** from three independent experiments. Results in **(A)** are expressed as mean ± SD, and results in **(C)** are expressed as mean ± SEM.

Steroidogenesis is a coordinated process regulated by signals from ovarian cells and is essential for steroid hormone production, which supports follicle development, oocyte maturation, and ovulation. Genes involved in steroidogenesis include steroidogenic acute regulatory protein (*Star*), cytochrome P450 side-chain cleavage enzyme (*Cyp11a1*), and anti-Müllerian hormone (*Amh*). FSHR regulates folliculogenesis; PER2 and CRY1 regulate CR; CYCLIN D2 promotes cell division; and p27 inhibits follicular cell proliferation. AMH is produced by granulosa cells of developing follicles and plays a role in controlling estrogen secretion. AMH levels are closely associated with the number of antral follicles and serve as a reliable indicator of ovarian reserve (27). To investigate the potential prophylactic and therapeutic effects of SR9011 (**Figures 2**, **3**) on ovarian steroidogenesis, follicular development, clock gene expression, and cell division in mice with disrupted CR, we measured the relative mRNA levels of *Amh*, *Star*, *Cyp11a1*, *Per2*, *Cry1*, and *FSHR* in control mice, CR-disrupted mice, and CR-disrupted mice treated with SR9011. These measurements were then correlated with the state of follicle development. mRNA expression of these genes was decreased in the ovaries of CR-disrupted mice compared with controls, indicating compromised follicular health and disrupted regulation of clock genes. In contrast, treatment with SR9011 in CR-disrupted mice elevated/restored the expression of genes associated with steroidogenesis, folliculogenesis, and cell division (**Figures 2A**, **3A**). However, therapeutic treatment did not significantly restore the expression of clock genes; other genes, such as *Amh*, *CYP11A1*, *Star*, and *FSHR*, were rescued by SR9011 treatment (**Figure 3A**). A subtle change was observed in *Per2* and *Cry1* expression at the mRNA level, whereas significant changes were observed at the protein level (**Figures 3B, C**).

Ovaries from CR-disrupted mice exhibited elevated p27 and reduced FSHR and CYCLIN D2 expression, indicative of impaired cell proliferation and increased follicular atresia, a trend confirmed by Western blot analyses (**Figures 2B, C**, **3B, C**). Notably, SR9011 treatment decreased p27 levels and increased the expression of CYP11A1, STAR, CRY1, PER2, FSHR, and CYCLIN D2 compared with CR-disrupted mice, suggesting restoration of cell proliferation, steroidogenesis, and follicle development.

### Prophylactic and therapeutic effects of SR9011 on follicle atresia and impaired follicle development due to chronodisruption

**Figure 4:**
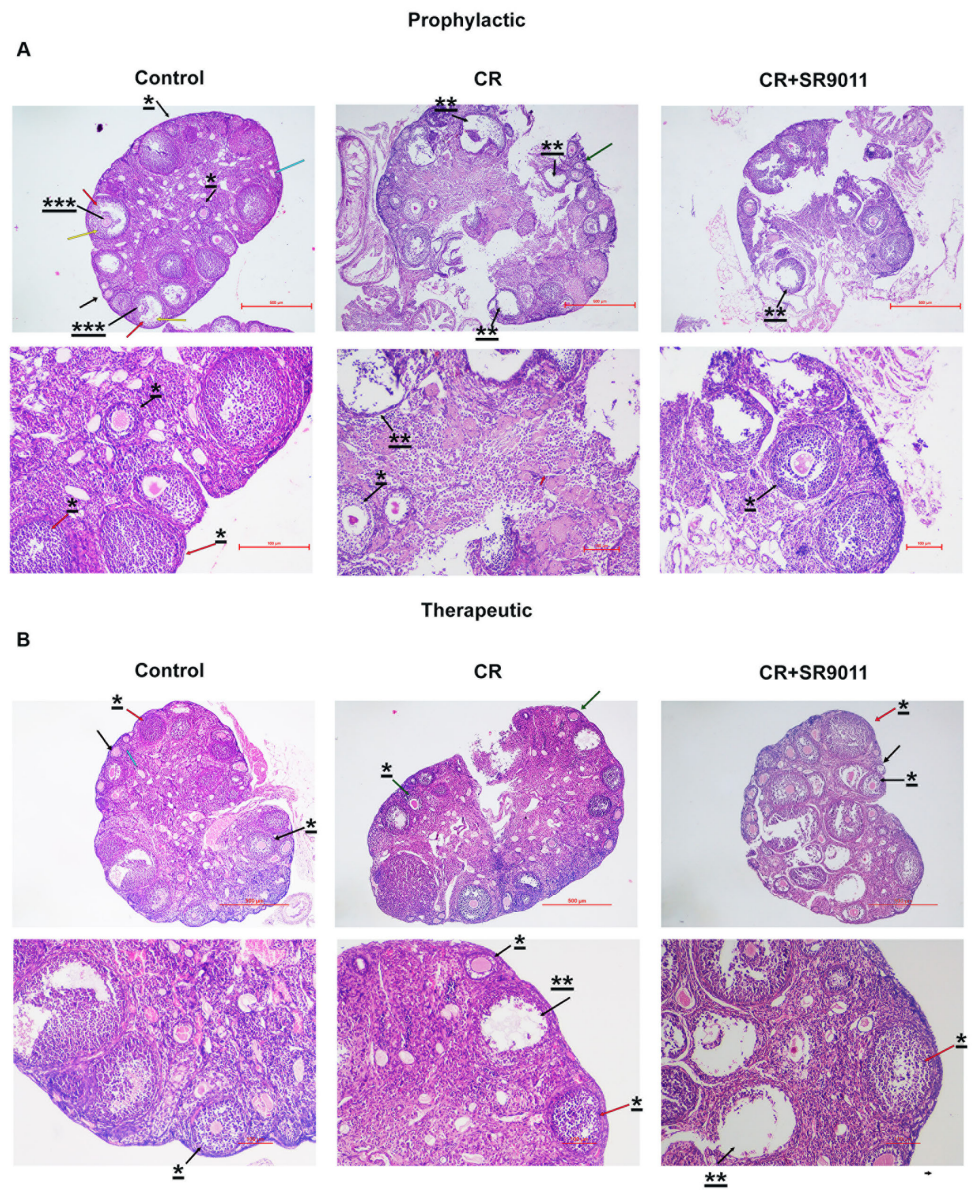
Effect of Rev-erbα on follicular growth and development. Hematoxylin and eosin staining was performed on ovarian sections from control mice, CR-disrupted mice, and CR-disrupted mice treated with SR9011. Treatment with SR9011 in both prophylactic **(A)** and therapeutic **(B)** modes effectively inhibited follicle degeneration caused by CR disruption. Data shown are representative images **(A, B)** from five mice in each group. Representative mouse ovary sections illustrate different stages of follicular development. A black arrow indicates a primary follicle. A black arrow marked with one asterisk (^*^) denotes a secondary follicle. A red arrow highlights the fluid-filled antral cavity. An arrow marked with three asterisks (^***^) indicates the oocyte. A yellow arrow denotes a Graafian (mature) follicle. A red arrow marked with one asterisk (^*^) indicates the corpus luteum. A black arrow marked with two asterisks (^**^) indicates a large antral follicle undergoing atresia, characterized by apoptotic granulosa cells. A blue arrow indicates the follicular cells. Representative ovarian sections were acquired at × 20 magnification (scale bar: 100 µm) and × 4 magnification (scale bar: 500 µm).

We next performed H&E staining to examine ovarian follicles at different developmental stages in the ovaries of control mice, CR-disrupted mice, and CR-disrupted mice treated with SR9011. We assessed both the prophylactic and therapeutic effects of SR9011 on follicular development and atresia (**Figures 4A, B**). CR-disrupted mouse ovaries exhibited fewer healthy follicles and a significantly higher rate of follicular atresia (atretic follicles) compared with control ovaries, which contained healthy and Graffian follicles at all developmental stages. Treatment with SR9011 reduced the number of atretic follicles and increased the presence of healthy follicles in CR-disrupted ovaries, indicating that SR9011 restores follicle quality impaired by CR disruption.

### Prophylactic and therapeutic effects of SR9011 on impaired granulosa cell proliferation and ovarian follicle development

**Figure 5:**
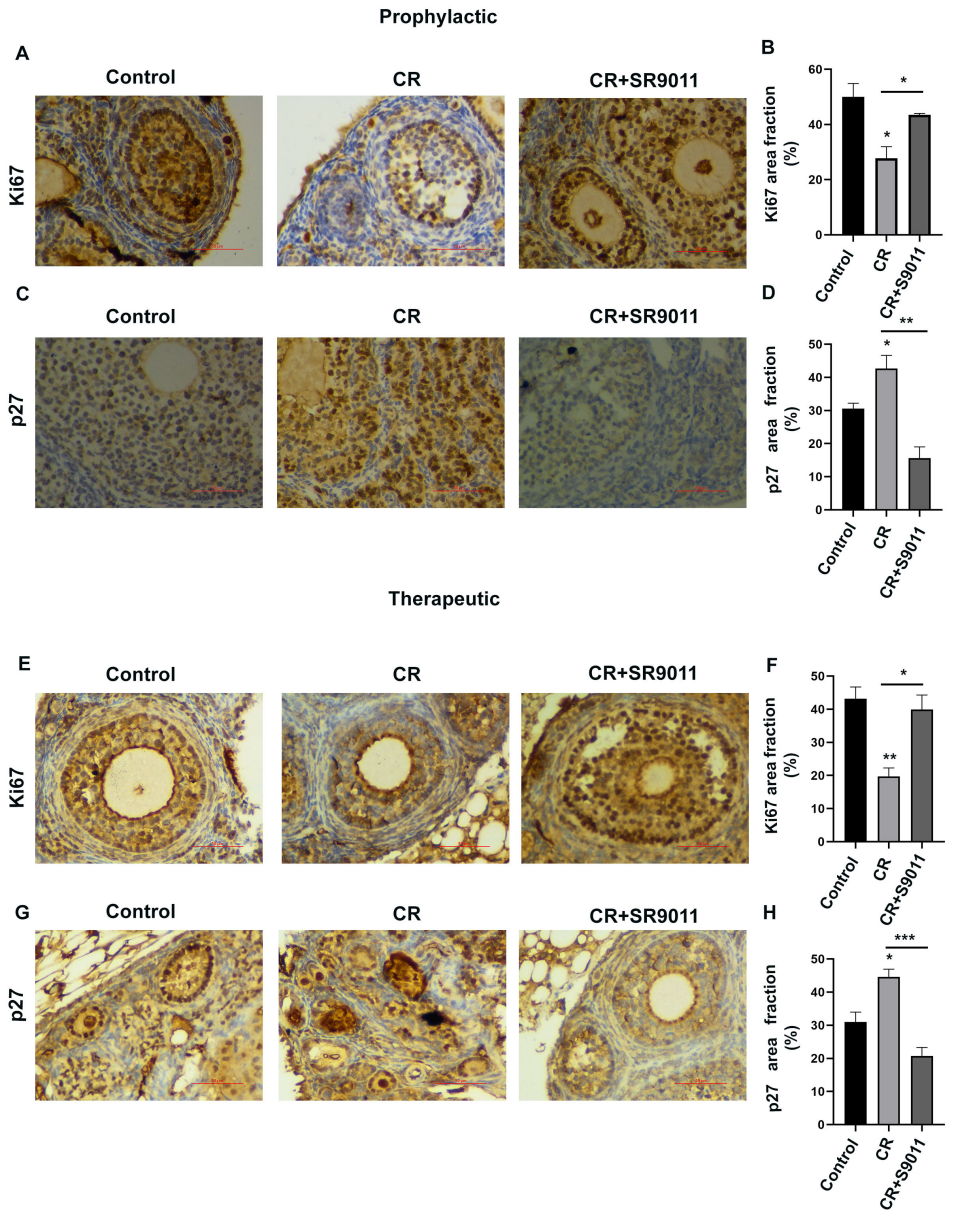
Effect of Rev-erbα on ovarian follicle development. Immunohistochemical detection of Ki67 and p27 was performed in ovarian sections from control mice, CR-disrupted mice, and CR-disrupted mice treated with SR9011. The use of SR9011 in prophylactic **(A–D)** and therapeutic **(E–H)**modes restored follicular growth impaired by CR disruption and promoted follicular cell proliferation. This effect is supported by increased Ki67 expression and decreased p27 expression compared with CR disruption alone. Data shown are representative images **(A, C, E, G)** from five mice in each group. Ki67 and p27 images were acquired at × 40 magnification (scale bar: 50 µm) in both the prophylactic and therapeutic groups. Graphs shown **(B, D, F, H)** are representative of different ovarian sections analyzed for quantification of the percentage area fraction of Ki67 and p27 staining (mean ± SEM). Asterisks represent significant differences as compared to control or as indicated (*** indicates P<0.001, ** indicates P<0.01, * indicates P<0.05 or P=0.05).

The reduced number of follicles in the ovaries of CR-disrupted mice suggests potential deficiencies in ovarian cell proliferation. To further evaluate the effects of SR9011 on follicular cell growth and division, we examined the expression of MKI67 (Ki67) and CDKN1B (p27) by immunohistochemistry in ovaries from control mice, CR-disrupted mice, and CR-disrupted mice treated with SR9011. Female mice received SR9011 (100 mg/kg) either during or after circadian disruption to assess the prophylactic (**Figures 5A–D**) and therapeutic effects (**Figures 5E–H**) of the ligand. Ovaries from the CR-disrupted mice exhibited a marked reduction in Ki67 expression, accompanied by increased expression of p27 in follicular cells (**Figures 5A–H**). Interestingly, SR9011 treatment restored follicle growth and promoted follicular cell proliferation and division, as evidenced by increased Ki67 expression and decreased p27 expression.

### Effect of SR9011 on follicle quantification after chronodisruption

**Figure 6:**
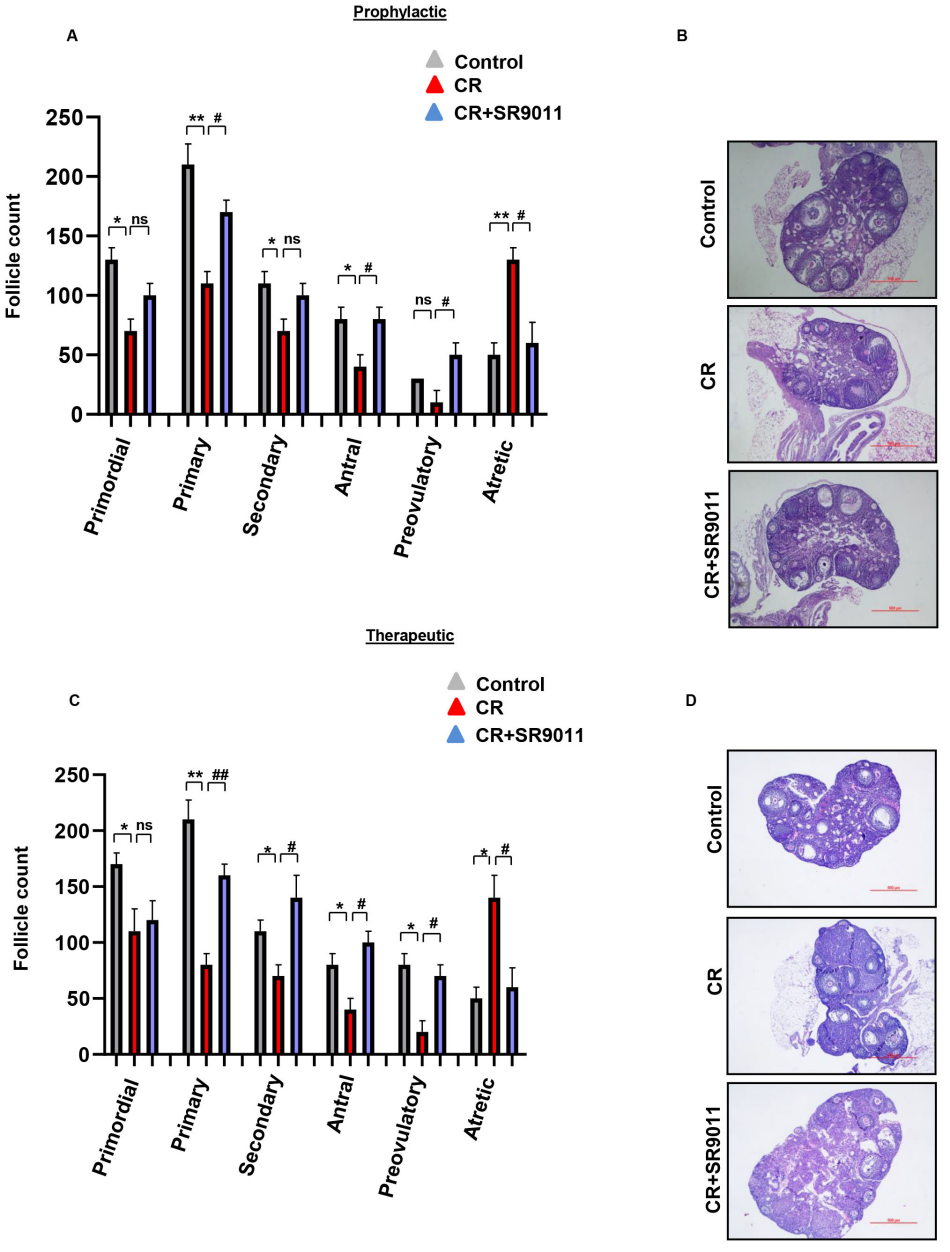
Effect of SR9011 on follicle quantification. Ovarian sections from control mice, CR-disrupted mice, and CR-disrupted mice treated with SR9011 were subjected to hematoxylin and eosin staining to assess follicle numbers at each stage of development. Treatment with SR9011 under prophylactic **(A, B)** and therapeutic **(C, D)** conditions restored follicular growth and reduced the number of atretic follicles compared with CR disruption alone. Asterisks represent significant differences compared with the control group, and hash symbols indicate significant differences compared with the CR-disrupted group (^**/##^*p* < 0.01, ^*/#^*p* < 0.05 or *p* = 0.05). Data are presented as the average **(A, C)** from three independent experiments (mean ± SEM) and as representative images **(B, D)** from three mice in each group.

Quantification of ovarian follicles at different developmental stages is a key indicator of folliculogenesis. **Figure 6** illustrates the observed primordial, primary, secondary, antral, preovulatory, and atretic follicles in the ovaries of control mice, CR-disrupted mice, and CR-disrupted mice treated with SR9011. As shown in **Figures 6A, C**, CR disruption affects both the quality and quantity of follicles (primordial, primary, secondary, antral, and preovulatory) and increases the number of atretic (degenerate) follicles. In contrast, SR9011 treatment, in both the prophylactic and therapeutic modes, improved follicle quality and increased follicle counts compared with CR disruption alone. Representative images are shown in **Figures 6B, D**.

### Effect of SR9011 on circadian desynchrony and hormone secretion

**Figure 7:**
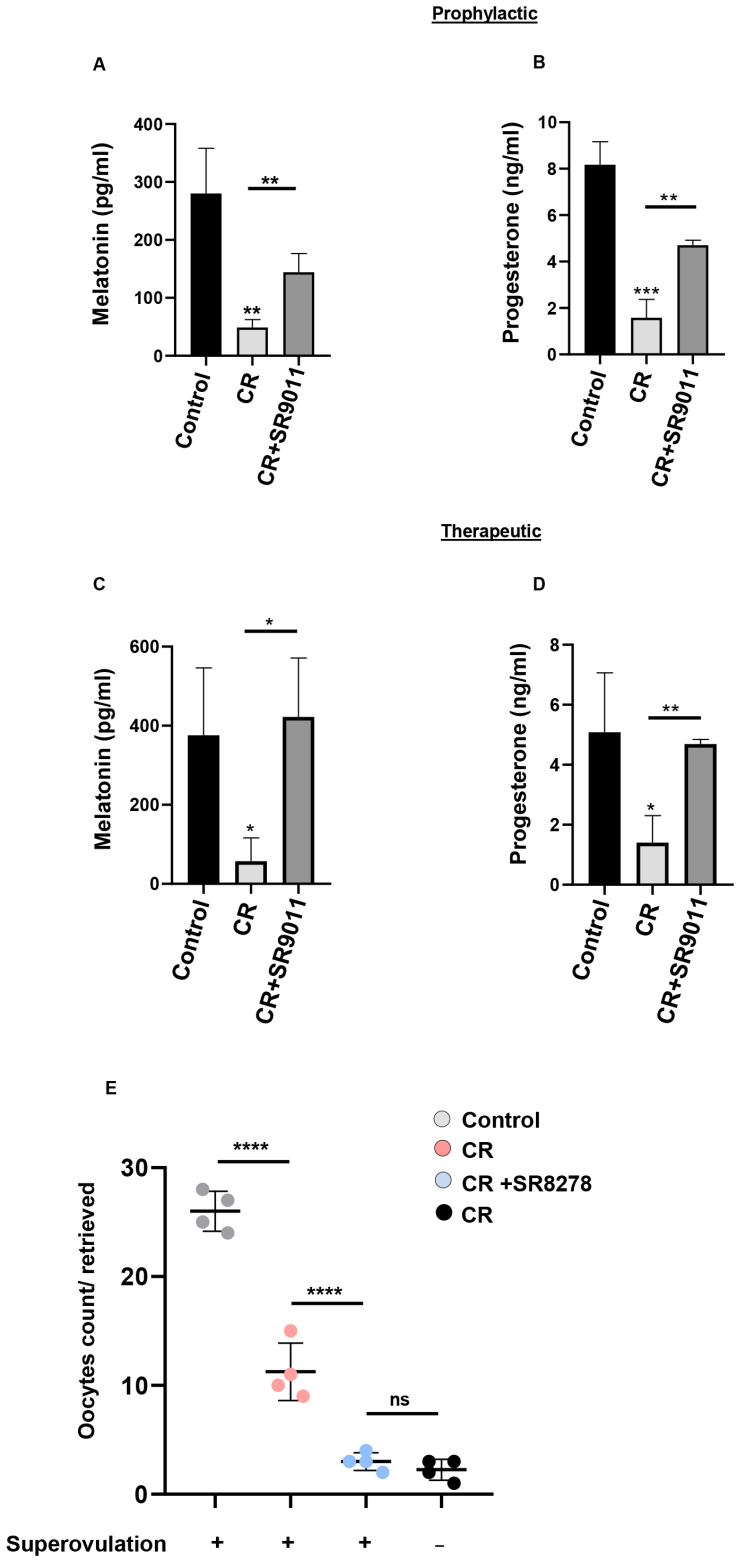
Rev-erbα: a key regulator of progesterone and melatonin synthesis and oocyte retrieval. The efficacy of SR9011 on melatonin and progesterone secretion following CR disruption was evaluated using both prophylactic **(A, B)** and therapeutic **(C, D)** approaches. Competitive ELISA was performed on serum samples isolated from control mice, CR-disrupted mice, and CR-disrupted mice treated with SR9011 to measure melatonin **(A, C)** and progesterone **(B, D)** levels. **(E)** Oocytes were collected from the oviducts and manually counted following superovulation. Each data point represents the oocyte yield of an individual mouse within the respective groups, with the horizontal line indicating the mean value for each group. The number of mice included in each group was as follows: control (*n* = 4), CR-disrupted only (*n* = 4), CR-disrupted + superovulation (*n* = 4), and CR-disrupted + SR8278 + superovulation (*n* = 4). Data are presented as the averages **(A–D)** from three independent experiments or from four mice in each group (mean ± SD). Asterisks represent significant differences as compared to control or as indicated (**** indicates P<0.0001, *** indicates P<0.001, ** indicates P<0.01, * indicates P<0.05 or P=0.05), and ns shows non-significant.

Circadian misalignment might have an impact on hormonal shifts during pregnancy, although the precise mechanisms underlying this phenomenon remain largely unclear. Pregnancy is closely linked to patterns of hormone secretion (28) as well as to the regulation of the sleep–wake cycle. Progesterone, a crucial hormone for maintaining pregnancy, reaches its peak levels around gestation days 15–17 in mice (29, 30). The sleep–wake cycle represents the most overt circadian rhythm. Melatonin, because of its rhythmic and cyclical release, plays a key role as a physiological regulator of the sleep–wake cycles in diurnal species, including humans. Melatonin is also synthesized in peripheral reproductive tissues, including cumulus oophorus, granulosa cells, and oocytes. Melatonin is a potent antioxidant that protects oocytes from oxidative stress, particularly during ovulation (31). Disruption of circadian rhythms can influence the secretion of this hormone (31). In this study, we measured serum levels of progesterone and melatonin in three distinct groups: control mice, CR-disrupted mice, and CR-disrupted mice treated with SR9011, to evaluate the impact of CR disruption and the efficacy of SR9011 in both prophylactic (**Figures 7A, B**) and therapeutic settings (**Figures 7C, D**). Compared with control mice, CR-disrupted mice exhibited reduced levels of both progesterone and melatonin. Treatment with SR9011 in CR-disrupted mice restored the levels of these hormones (**Figures 7A–D**).

To determine whether infertility was due to a defect in Rev-erbα or other secondary changes, we performed a superovulation experiment following a standard protocol. Mice were divided into four groups, with three groups undergoing superovulation induction. The fourth group was treated with SR8278 (14 mg/kg) on alternate days for 4 weeks. Forty-eight hours after the last SR8278 injection, superovulation was performed in the three relevant groups, except for the CR-disrupted group. The number of oocytes released into the oviduct was counted in the control (superovulated), CR (without superovulation), CR (superovulated), and CR + SR8278 (superovulated) groups.

As shown in **Figure 7E**, control females ovulated an average of 26 ± 1.82 oocytes following superovulation. In contrast, CR-disrupted females without superovulation released an average of 2.25 ± 0.95 oocytes, while CR-disrupted females undergoing superovulation released 11.25 ± 2.62 oocytes. These results indicate that oocyte retrieval in CR-disrupted females occurs primarily after exogenous gonadotropin administration. Interestingly, CR-disrupted mice treated with SR8278 released only 3 ± 0.81 oocytes following superovulation, a number comparable to CR-disrupted females without superovulation. This finding suggests that inhibiting Rev-erbα impairs oocyte retrieval, even when superovulation is performed.

## Discussion

The decline in female fertility has emerged as a major global health concern, and growing evidence suggests that circadian rhythms play a crucial role in regulating reproductive function in both animals and humans (32, 33). Beyond regulating the sleep–wake cycle, the circadian clock coordinates endocrine, metabolic, and proliferative processes essential for reproductive cyclicity. Disruptions of these rhythms—by shift work, poor diet, irregular light exposure, and chronic stress—negatively impact female reproductive health.

In this study, constant darkness was used to induce chronodisruption in mice. While this approach allows free-running rhythms, the persistence of rhythmicity under these conditions indicates endogenous circadian control rather than direct responsiveness to light–dark cues. Prolonged removal of environmental entrainment is known to induce internal circadian desynchrony, particularly between central and peripheral clocks. Maintaining mice in constant darkness for 4–6 weeks was sufficient to induce molecular circadian desynchrony in the ovary. Our focus was on ovarian circadian integrity rather than behavioral rhythmicity, and disruption was validated at the molecular and functional levels by reduced ovarian expression of core clock genes (*Rev-erbα*, *Per2*, *Cry1*), decreased circulating melatonin, and impaired circadian-regulated reproductive processes, including steroidogenesis, folliculogenesis, and fertility. These findings support the concept that ovarian physiology is tightly coupled to an intact peripheral circadian clock.

Emerging evidence indicates that circadian clock components play a critical role in ovarian function by coordinating transcriptional programs that regulate granulosa cell proliferation, metabolism, and endocrine activity. PER2 and CLOCK regulate steroid hormone production and granulosa cell proliferation (34), and molecular circadian clock genes are expressed in human luteinized granulosa cells, with reduced expression of some genes observed in older women, which may partly contribute to age-related fertility decline (35). However, the mechanistic contribution of individual circadian regulators to ovarian dysfunction under chronodisruption has not been adequately defined.

Rev-erbα is a core circadian nuclear receptor that functions as a transcriptional repressor via NCoR/HDAC3 recruitment (36). In addition to maintaining circadian rhythmicity, Rev-erbα integrates circadian timing with tissue-specific metabolic and transcriptional programs. In the ovary, Rev-erbα exhibits rhythmic expression in granulosa cells and tumors (37–39). Importantly, Rev-erbα functions in steroid biosynthesis by regulating STAR and PER2 (40), positioning it as a molecular node connecting clock machinery with reproductive output. It also inhibits apoptosis in granulosa cells (41) and maintains autophagy homeostasis by suppressing ATG5 (42), thereby preventing follicular atresia.

Our findings demonstrate that circadian disruption suppresses core clock components, including *Rev-erbα*, *Per2*, and *Cry1*, which coincides with impaired granulosa cell proliferation, reduced steroidogenic capacity, increased follicular atresia, and diminished fertility. PER2 and CRY1 are central components of the molecular circadian clock that coordinate ovarian cell metabolism, proliferation, and steroidogenesis. The observed reduction of these proteins under CR disruption suggests that loss of circadian timing directly impairs granulosa cell function and follicular competence. Specifically, elevated expression of the cell-cycle inhibitor p27 (CDKN1B) and reduced expression of CYCLIN D2, FSHR, and AMH indicate a shift of granulosa cells toward a quiescent, atresia-prone state rather than active follicular growth. p27 enforces cell-cycle arrest, whereas CYCLIN D2 is essential for granulosa cell proliferation, follicular expansion, and ovulation. These alterations provide a mechanistic basis for the observed depletion of healthy follicles under circadian disruption.

In parallel, reduced expression of the steroidogenic genes STAR and CYP11A1 indicates impaired cholesterol transport into mitochondria and diminished progesterone biosynthesis—key processes essential for follicle maturation and luteal function.

Pharmacological activation of Rev-erbα restored PER2 and CRY1 expression, accompanied by improved expression of steroidogenic enzymes (STAR, CYP11A1), folliculogenic markers, and cell-cycle regulators (increased CYCLIN D2 and decreased p27), resulting in reduced follicular atresia and enhanced reproductive outcomes. These findings indicate that Rev-erbα supports ovarian function by maintaining PER–CRY balance, thereby linking circadian timing to reproductive competence. Notably, although the recovery of clock gene transcripts was modest, restoration at the protein level suggests that Rev-erbα may exert downstream regulatory effects on granulosa cell transcriptional networks independent of a complete circadian transcriptional reset.

Melatonin, a key circadian hormone secreted by the pineal gland, synchronizes the molecular clock, particularly in the ovary, through interactions with clock genes. Dysregulation of melatonin signaling disrupts ovarian circadian rhythms, contributing to impaired steroidogenesis, follicular development, and overall female reproductive pathophysiology. Human granulosa cells express melatonin and its receptors, which modulate basal progesterone production (43). In our study, melatonin and progesterone levels were significantly reduced following chronodisruption, whereas activation of Rev-erbα with SR9011 restored both circulating melatonin and progesterone, indicating that Rev-erbα helps maintain hormonal homeostasis under circadian stress.

Finally, the SR9011 dose was selected based on our previous studies in mice, in which this concentration effectively modulated Rev-erbα activity (36, 44). The functional importance of Rev-erbα was further supported by pharmacological antagonism using SR8278, which markedly impaired ovarian responsiveness to exogenous gonadotropins and reduced oocyte retrieval, even after superovulation. These findings reinforce the direct requirement for Rev-erbα activity in preserving granulosa cell competence, follicular reserve, and ovarian responsiveness under conditions of circadian misalignment. Collectively, these results demonstrate that Rev-erbα integrates circadian signals with ovarian function, maintaining follicular health, steroidogenesis, and fertility.

Taken together, our results position Rev-erbα as a key integrator of female infertility associated with tissue-level circadian misalignment. Although continuous circadian profiling of PER, CRY, and melatonin would provide more detailed rhythmicity, our single-time-point molecular and endocrine measurements sufficiently demonstrate circadian misalignment in the ovary and validate the restorative effects of SR9011.

In conclusion, Rev-erbα plays a pivotal role in coordinating ovarian circadian signals, steroidogenesis, and follicular integrity, providing a potential avenue for therapeutic intervention in female infertility resulting from circadian disruption.

## Data Availability

The mandatory gene expression data are provided as **Supplementary files** (Gene expression data files 1-9). All data generated and analyzed during the study have also been deposited with the Institute of Microbial Technology data repository. Further inquiries about data access can be obtained from corresponding author upon request.

## Supplementary material

Supplementary Figure 1 Impact of Circadian rhythm disruption on mice fertility. The breeding efficiency was deciphered to show how circadian rhythm affects reproductive performance of control pairs and CR-disrupted pairs. Control pairs and CR-disrupted pairs shown in both prophylactic (A and B resp.) and therapeutic (C and D resp.). The x-axis demonstrates the elapsed numbers of days since the mating trial commenced, with each litter represented by a vertical axis corresponding to the cumulative number of offspring produced. The graph illustrates data from individual cages, showing **(A)** a control pair that produced 12 live litters within a span of 362 days, and **(B)** a pair with disrupted CR, resulting in 8 live litters over 363 days. **(C)** a control pair that produced 10 live litters over a period of 388 days **(D)** CR-disrupted pair produced 8 live litters over 345 days. Mean litter interval was presented as (mean time interval between litters ± sd).

Supplementary Figure 2 Rev-erbα in chrono-disruption. qRT-PCR analysis to check the expression of *Rev-erbα*, *Per2* and *Cry1* in control and CR-disrupted mice ovaries. Asterisks represent significant differences as compared to control or as indicated (**** indicates *P* < 0.0001, *** indicates *P* < 0.001, ** indicates *P* < 0.01, * indicates *P* < 0.05). Data shown are average from three independent experiments (mean ± sd).

## References

[1] Gupta S Gupta P . Etiopathogenesis, challenges and remedies associated with female genital tuberculosis: potential role of nuclear receptors. Front Immunol. (2020) 11:2161. doi: 10.3389/fimmu.2020.02161, PMID: 33178178 PMC7593808

[2] Sciarra F Franceschini E Campolo F Gianfrilli D Pallotti F Paoli D . Disruption of circadian rhythms: A crucial factor in the etiology of infertility. IJMS. (2020) 21:3943. doi: 10.3390/ijms21113943, PMID: 32486326 PMC7312974

[3] Kloss JD Perlis ML Zamzow JA Culnan EJ Gracia CR . Sleep, sleep disturbance, and fertility in women. Sleep Med Rev. (2015) 22:78–87. doi: 10.1016/j.smrv.2014.10.005, PMID: 25458772 PMC4402098

[4] Voigt RM Forsyth CB Keshavarzian A . Circadian disruption: potential implications in inflammatory and metabolic diseases associated with alcohol. Alcohol Res. (2013) 35:87–96. doi: 10.35946/arcr.v35.1.10, PMID: 24313168 PMC3860420

[5] Parua S Choudhury GR Bhattacharya S Hazra A Dutta S Sengupta P . Melatonin in female fertility: multifaceted role from reproductive physiology to therapeutic potential in polycystic ovary syndrome, endometriosis, and ovarian failure. Chronobiol Med. (2024) 6:145–62. doi: 10.33069/cim.2024.0022

[6] Chen W Zhang H Guo B Tao Y Zhang J Wang J . Melatonin refines ovarian mitochondrial dysfunction in PCOS by regulating the circadian rhythm gene Clock. Cell Mol Life Sci. (2025) 82:104. doi: 10.1007/s00018-025-05609-9, PMID: 40047877 PMC11885701

[7] Tao J Zhang L Zhang X Chen Y Chen Q Shen M . Effect of exogenous melatonin on the development of mice ovarian follicles and follicular angiogenesis. IJMS. (2021) 22:11262. doi: 10.3390/ijms222011262, PMID: 34681919 PMC8540648

[8] Vasquez YM DeMayo FJ . Role of nuclear receptors in blastocyst implantation. Semin Cell Dev Biol. (2013) 24:724–35. doi: 10.1016/j.semcdb.2013.08.004, PMID: 23994285 PMC3849351

[9] Bertolin K Gossen J Schoonjans K Murphy BD . The orphan nuclear receptor nr5a2 is essential for luteinization in the female mouse ovary. Endocrinology. (2014) 155:1931–43. doi: 10.1210/en.2013-1765, PMID: 24552399

[10] Duggavathi R Volle DH Mataki C Antal MC Messaddeq N Auwerx J . Liver receptor homolog 1 is essential for ovulation. Genes Dev. (2008) 22:1871–6. doi: 10.1101/gad.472008, PMID: 18628394 PMC2492734

[11] Kin Ting Kam R Deng Y Chen Y Zhao H . Retinoic acid synthesis and functions in early embryonic development. Cell Biosci. (2012) 2:11. doi: 10.1186/2045-3701-2-11, PMID: 22439772 PMC3325842

[12] Pereira FA Qiu Y Zhou G Tsai M-J Tsai SY . The orphan nuclear receptor COUP-TFII is required for angiogenesis and heart development. Genes Dev. (1999) 13:1037–49. doi: 10.1101/gad.13.8.1037, PMID: 10215630 PMC316637

[13] Petit FG Jamin SP Kurihara I Behringer RR DeMayo FJ Tsai M-J . Deletion of the orphan nuclear receptor COUP-TFII in uterus leads to placental deficiency. Proc Natl Acad Sci USA. (2007) 104::6293–6298. doi: 10.1073/pnas.0702039104, PMID: 17404209 PMC1851059

[14] Jeyasuria P Ikeda Y Jamin SP Zhao L De Rooij DG Themmen APN . Cell-specific knockout of steroidogenic factor 1 reveals its essential roles in gonadal function. Mol Endocrinol. (2004) 18:1610–9. doi: 10.1210/me.2003-0404, PMID: 15118069

[15] Pelusi C Ikeda Y Zubair M Parker KL . Impaired follicle development and infertility in female mice lacking steroidogenic factor 1 in ovarian granulosa cells. Biol Reprod. (2008) 79:1074–83. doi: 10.1095/biolreprod.108.069435, PMID: 18703422 PMC2780474

[16] Cloke B Christian M . The role of androgens and the androgen receptor in cycling endometrium. Mol Cell Endocrinol. (2012) 358:166–75. doi: 10.1016/j.mce.2011.06.031, PMID: 21745536

[17] Lobaccaro JMA Gallot D Lumbroso S Mouzat. Liver X Receptors K . and female reproduction: When cholesterol meets fertility! J Endocrinological Invest. (2013) 36:55–60. doi: 10.3275/8765, PMID: 23211426

[18] Shahbazi M Jeddi-Tehrani M Zareie M Salek-Moghaddam A Akhondi MM Bahmanpoor M . Expression profiling of vitamin D receptor in placenta, decidua and ovary of pregnant mice. Placenta. (2011) 32:657–64. doi: 10.1016/j.placenta.2011.06.013, PMID: 21764449

[19] Irani M Merhi Z . Role of vitamin D in ovarian physiology and its implication in reproduction: a systematic review. Fertility Sterility. (2014) 102:460–468.e3. doi: 10.1016/j.fertnstert.2014.04.046, PMID: 24933120

[20] Zhao X Cho H Yu RT Atkins AR Downes M Evans RM . Nuclear receptors rock around the clock. EMBO Rep. (2014) 15:518–28. doi: 10.1002/embr.201338271, PMID: 24737872 PMC4210094

[21] Mang GM La Spada F Emmenegger Y Chappuis S Ripperger JA Albrecht U . Altered sleep homeostasis in rev-erb α Knockout mice. Sleep. (2016) 39:589–601. doi: 10.5665/sleep.5534, PMID: 26564124 PMC4763348

[22] Amador A Huitron-Resendiz S Roberts AJ Kamenecka TM Solt LA Burris TP . Pharmacological targeting the REV-ERBs in sleep/wake regulation. PloS One. (2016) 11:e0162452. doi: 10.1371/journal.pone.0162452, PMID: 27603791 PMC5014418

[23] Wang S Li F Lin Y Wu B . Targeting REV-ERBα for therapeutic purposes: promises and challenges. Theranostics. (2020) 10:4168–82. doi: 10.7150/thno.43834, PMID: 32226546 PMC7086371

[24] Handelsman DJ Walters KA Ly LP . Simplified method to measure mouse fertility. Endocrinology. (2020) 161:bqaa114. doi: 10.1210/endocr/bqaa114, PMID: 32645712

[25] Tilly JL . Commuting the death sentence: how oocytes strive to survive. Nat Rev Mol Cell Biol. (2001) 2:838–48. doi: 10.1038/35099086, PMID: 11715050

[26] Hirshfield AN Rees Midgley A . Morphometric analysis of foilicular development in the rat. Biol Reprod. (1978) 19:597–605. doi: 10.1095/biolreprod19.3.597, PMID: 363183

[27] Dewailly D Laven J . AMH as the primary marker for fertility. Eur J Endocrinol. (2019) 181:D45–51. doi: 10.1530/EJE-19-0373, PMID: 31398713

[28] Kumar P Magon N . Hormones in pregnancy. Niger Med J. (2012) 53:179. doi: 10.4103/0300-1652.107549, PMID: 23661874 PMC3640235

[29] Barkley MS Michael SD Geschwind II Bradford GE . Plasma testosterone during pregnancyin the mouse. Endocrinology. (1977) 100:1472–5. doi: 10.1210/endo-100-5-1472, PMID: 849739

[30] Virgo BB Bellward GD . Serum progesterone levels in the pregnant and postpartum laboratory mouse. Endocrinology. (1974) 95:1486–90. doi: 10.1210/endo-95-5-1486, PMID: 4473330

[31] Reiter RJ Tamura H Tan DX Xu X-Y . Melatonin and the circadian system: contributions to successful female reproduction. Fertility Sterility. (2014) 102:321–8. doi: 10.1016/j.fertnstert.2014.06.014, PMID: 24996495

[32] Chen M Xu Y Miao B Zhao H Luo L Shi H . Expression pattern of circadian genes and steroidogenesis-related genes after testosterone stimulation in the human ovary. J Ovarian Res. (2016) 9:56. doi: 10.1186/s13048-016-0264-5, PMID: 27614897 PMC5018165

[33] Srinivasan V Spence WD Pandi-Perumal SR Zakharia R Bhatnagar KP Brzezinski A . Melatonin and human reproduction: shedding light on the darkness hormone. Gynecol Endocrinol. (2009) 25:779–85. doi: 10.3109/09513590903159649, PMID: 19905996

[34] Shimizu T Hirai Y Murayama C Miyamoto A Miyazaki H Miyazaki K . Circadian Clock genes Per2 and clock regulate steroid production, cell proliferation, and luteinizing hormone receptor transcription in ovarian granulosa cells. Biochem Biophys Res Commun. (2011) 412:132–5. doi: 10.1016/j.bbrc.2011.07.058, PMID: 21819971

[35] Brzezinski A Saada A Miller H Brzezinski-Sinai NA Ben-Meir A . Is the aging human ovary still ticking?: Expression of clock-genes in luteinized granulosa cells of young and older women. J Ovarian Res. (2018) 11:95. doi: 10.1186/s13048-018-0471-3, PMID: 30463623 PMC6247686

[36] Gupta S Ahuja N Kumar S Arora R Kumawat S Kaushal V . Rev-erbα regulate neurogenesis through suppression of Sox2 in neuronal cells to regenerate dopaminergic neurons and abates MPP+ induced neuroinflammation. Free Radical Biol Med. (2024) 223:144–59. doi: 10.1016/j.freeradbiomed.2024.07.025, PMID: 39084577

[37] Zhang J Zhao L Li Y Dong H Zhang H Zhang Y . Circadian clock regulates granulosa cell autophagy through NR1D1-mediated inhibition of ATG5. Am J Physiol Cell Physiol. (2022) 322:C231–45. doi: 10.1152/ajpcell.00267.2021, PMID: 34936504

[38] Alexiadis M Eriksson N Jamieson S Davis M Drummond AE Chu S . Nuclear receptor profiling of ovarian granulosa cell tumors. Horm Canc. (2011) 2:157–69. doi: 10.1007/s12672-011-0069-3, PMID: 21761343 PMC10358074

[39] Boden MJ Varcoe TJ Voultsios A Kennaway DJ . Reproductive biology of female Bmal1 null mice. Reproduction. (2010) 139:1077–90. doi: 10.1530/REP-09-0523, PMID: 20200203

[40] Chen H Chu G Zhao L Yamauchi N Shigeyoshi Y Hashimoto S . Rev-erbα regulates circadian rhythms and StAR expression in rat granulosa cells as identified by the agonist GSK4112. Biochem Biophys Res Commun. (2012) 420:374–9. doi: 10.1016/j.bbrc.2012.02.164, PMID: 22425774

[41] Sun L Tian H Xue S Ye H Xue X Wang R . Circadian clock genes REV-ERBs inhibits granulosa cells apoptosis by regulating mitochondrial biogenesis and autophagy in polycystic ovary syndrome. Front Cell Dev Biol. (2021) 9:658112. doi: 10.3389/fcell.2021.658112, PMID: 34422794 PMC8374745

[42] Zhang T Lin M Wang C Zhou J . Mechanisms of follicular atresia: focus on apoptosis, autophagy, and ferroptosis. Front Endocrinol (Lausanne). (2025) 16:1603467. doi: 10.3389/fendo.2025.1603467, PMID: 41064363 PMC12500450

[43] Fang L Li Y Wang S Yu Y Li Y Guo Y . Melatonin induces progesterone production in human granulosa-lutein cells through upregulation of StAR expression. Aging (Albany NY). (2019) 11:9013–24. doi: 10.18632/aging.102367, PMID: 31619582 PMC6834401

[44] Kumar S Arora R Gupta S Ahuja N Bhagyaraj E Nanduri R . Nuclear receptor Rev-erbα role in fine-tuning erythropoietin gene expression. Blood Adv. (2024) 8:3705–17. doi: 10.1182/bloodadvances.2023012228, PMID: 38748870 PMC11296239

