# Supplementary figures and images for: Ovarian Rev-erbα: a central regulator of fertility following chronodisruption
Source: Front Endocrinol (Lausanne). 2026 Feb 18;17:1742988. doi: 10.3389/fendo.2026.1742988 (PMC12957795; doi:10.3389/fendo.2026.1742988)

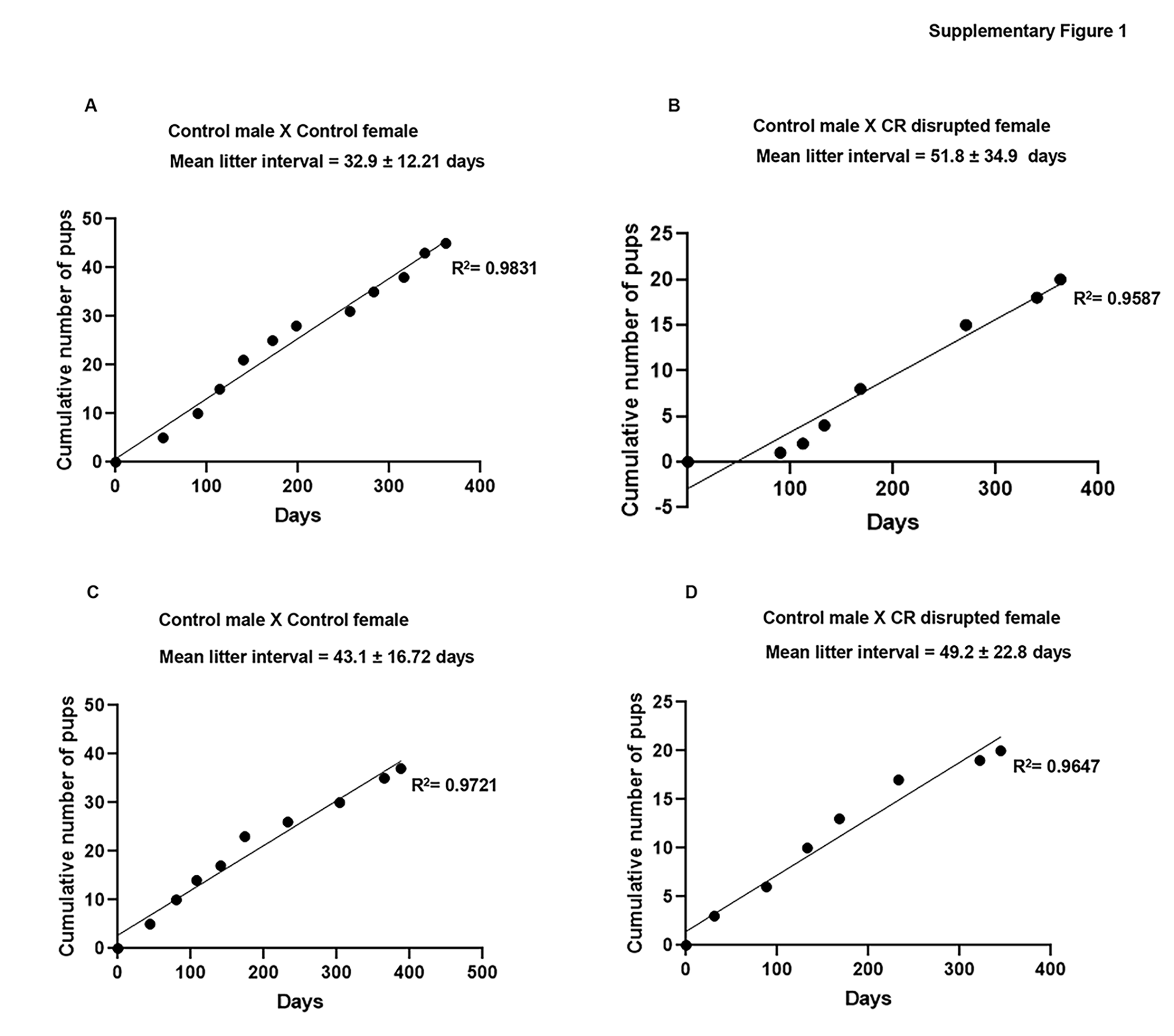

Supplement: Supplementary Figure 1 — Impact of Circadian rhythm disruption on mice fertility. The breeding efficiency was deciphered to show how circadian rhythm affects reproductive performance of control pairs and CR-disrupted pairs. Control pairs and CR-disrupted pairs shown in both prophylactic (A and B resp.) and therapeutic (C and D resp.). The x-axis demonstrates the elapsed numbers of days since the mating trial commenced, with each litter represented by a vertical axis corresponding to the cumulative number of offspring produced. The graph illustrates data from individual cages, showing (A) a control pair that produced 12 live litters within a span of 362 days, and (B) a pair with disrupted CR, resulting in 8 live litters over 363 days. (C) a control pair that produced 10 live litters over a period of 388 days (D) CR-disrupted pair produced 8 live litters over 345 days. Mean litter interval was presented as (mean time interval between litters ± sd). [file Image1.tif]

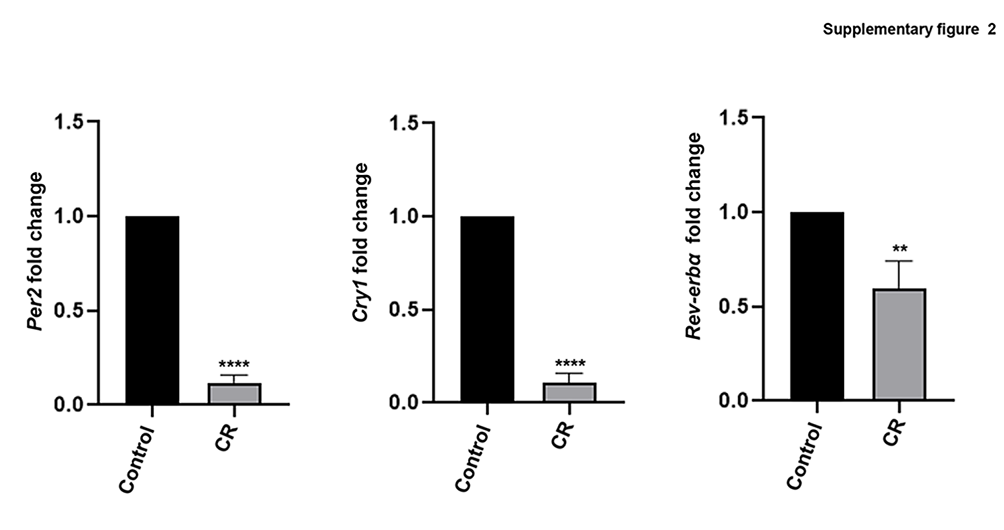

Supplement: Supplementary Figure 2 — Rev-erbα in chrono-disruption. qRT-PCR analysis to check the expression of Rev-erbα, Per2 and Cry1 in control and CR-disrupted mice ovaries. Asterisks represent significant differences as compared to control or as indicated (**** indicates P < 0.0001, *** indicates P < 0.001, ** indicates P < 0.01, * indicates P < 0.05). Data shown are average from three independent experiments (mean ± sd). [file Image2.tif]
